# Design and implementation of a Serious Game on neurorehabilitation: Data on modifications of functionalities along implementation releases

**DOI:** 10.1016/j.dib.2018.08.100

**Published:** 2018-08-30

**Authors:** Federica Savazzi, Sara Isernia, Johanna Jonsdottir, Sonia Di Tella, Stefania Pazzi, Francesca Baglio

**Affiliations:** a IRCCS Fondazione don Carlo Gnocchi ONLUS, Milan, Italy; b Università Cattolica del Sacro Cuore, Milan, Italy; c Consorzio di Bioingegneria e Informatica Medica -CBIM, Pavia, Italy

## Abstract

The measurement of users’ perception of functionalities in the use of Serious Games (SGs) along technology implementation phases may lead to effective changes for developing successful user-centered learning tools in the medical field. In the present data article, data about usability functionalities along two cycles of validation of a SG on neurorehabilitation with final users are described. The key principles of usability model used to collect and analyze data and the evaluation tool are presented. The modifications of the SG to improve usability across implementation phases are detailed. The validation of the SG is described in “Engaged in learning neurorehabilitation: development and validation of a serious game with user-centered design” (Savazzi et al., in press) [1]. The data provided in this article will assist researchers working for developing learning technology to optimize their tools in relation to users’ needs and expectations.

**Specifications Table**

**Table:** 

Subject area	*Psychology*
More specific subject area	*Serious Game implementation for education*
Type of data	*Table, text file, figure*
How data was acquired	*Ad hoc-questionnaire*
Data format	*Analyzed*
Experimental factors	*No pretreatment of samples was conducted*
Experimental features	*Application of the User Centered Design model for the implementation of serious game usability along implementation phases through an ad-hoc questionnaire*
Data source location	*Milan, Italy*
Data accessibility	*Data are with this article*

**Value of the data**

• *The data on Serious Game (SG) functionalities could be compared to data on other SGs developed with the User Centered Design model for further insight on SG implementation*
• *The data could serve as a benchmark for other researchers to assess the functionalities of an educational SG for improving usability*
• *The data could be used in the development of further experiments on development and validation of SGs*

## 1 Data

Serious Games (SGs) are technological tools with substantial effectiveness in learning. The present article reports data of the validation of a Serious Game (SG) on neurorehabilitation along two steps of implementation following the User Centered Design (UCD) model. In particular, the usability principles guiding the SG implementation, the tool adopted to collect data and the data obtained are described.

## 2 Experimental design, materials and methods

### 2.1 Procedure

We adopted a User Centered Design model to validate a neurorehabilitation-focused SG in order to monitor its usability and functionality along implementation phases. In particular, two cycles of *design-evaluation-redesign* were performed in which data on users experience provided informative feedback to make changes in the SG strictly related to users’ needs. In particular, data were collected and analyzed to reach a good level of SG usability. Accordingly, an adaptation of the Nielsen–Shneiderman usability principles [2] guided the modifications for SG implementation. Table 1 reports and briefly describes the Nielsen–Shneiderman usability principles [3], [4].

**Table 1:** Usability principles defined for the SG design and implementation.

	**Usability principle**^a^	**Definition**
***1***	***Consistency/Minimalism***	• Consistency throughout the SG in the font/capitalization, color/layout, and positioning is required. • Elements increasing perceived immersion (variation of types of tasks and questions) should be preferred to elements that distract or cause slowdown (too much text; too many features; and long and/or complex questions; too long scenarios).
***2***	***Visibility/Documentation***	• The SG should be instinctive to use. • Information on how to interact with the SG (how to play the SG, possibilities of action, and how to use the different features on the screen) should be provided. • Visibility may be influenced by colors on text and screens.
***3***	***Match***	• The SG and the real world should match. • Irrelevant information is to be avoided. • In the quiz-based tasks or questions, all answers should connect to the question and be plausible answers.
***4***	***Memory***	• Avoid the need for users to memorize a lot of information related to: a. how to play the SG should be avoided. b. the story in the SG.
***5***	***Feedback/Closure***	• Feedback about users’ actions and performance should be provided during the game and at the end of the game. • Debriefing is also suggested.
***6***	***Flexibility/Control/Undo***	• The interaction with the SG should allow flexibility (move back and forth in the SG; pause the game; undo answers; view own answers; distinguish right and wrong answers, view the consequences of entering wrong answers).
***7***	***Error/Message***	• Prevent errors in the SGs during design. • Provide messages/alerts on the screen about possible errors and how to recover from these errors.
***8***	***Language***	• Provide clear language and concepts understandable for the intended users.

a Based on Nielsen–Shneiderman usability heuristics adapted from Zhang and Walji [2].

### 2.2 Participants

In order to verify whether the key core usability principles (Table 1) were being followed during SG implementation, two groups of physiotherapists (*N* = 10; *N* = 28) experienced the tool in two different steps of development of the SG.

Ethical Committee of Don Gnocchi Foundation of Milan approved the data collection and all subjects involved received the information sheet and signed the written informed consent.

### 2.3 Materials

We administered to both groups a 10-item *ad-hoc* questionnaire on SG functionalities (Cronbach׳s alpha = .87). The final part of the questionnaire included two open-answer questions investigating users’ opinion on the game most difficult and most pleasant parts. The answers to the two final questions were categorized in eight usability principles (Table 1) by two independent judges. The items of the questionnaire are reported in Table 2.

**Table 2:** *Ad-hoc* questionnaire on SG functionalities.

**Item**	**Question**	**Response**				
1	I understood the scenario	*Totally disagree*	*Disagree*	*I don’t know*	*Agree*	*Totally agree*
2	I was able to identify the medical record	*Totally disagree*	*Disagree*	*I don’t know*	*Agree*	*Totally agree*
3	I understood the instructions	*Totally disagree*	*Disagree*	*I don’t know*	*Agree*	*Totally agree*
4	I could not have navigated through the game without the instructions	*Totally disagree*	*Disagree*	*I don’t know*	*Agree*	*Totally agree*
5	I will have to look for assistance often when I play the game	*Totally disagree*	*Disagree*	*I don’t know*	*Agree*	*Totally agree*
6	The game has an attractive presentation	*Totally disagree*	*Disagree*	*I don’t know*	*Agree*	*Totally agree*
7	Learning to use this game is easy	*Totally disagree*	*Disagree*	*I don’t know*	*Agree*	*Totally agree*
8	The control of the game is intuitive	*Totally disagree*	*Disagree*	*I don’t know*	*Agree*	*Totally agree*
9	It was generally easy to play the game	*Totally disagree*	*Disagree*	*I don’t know*	*Agree*	*Totally agree*
10	The game fulfilled the described rules	*Totally disagree*	*Disagree*	*I don’t know*	*Agree*	*Totally agree*
11	What was the most difficult part to understand?	*Free text*				
12	What did you like the most while playing the game?	*Free text*				

### 2.4 Data

Data collected for the first ten items showed high mean responses (Means > 3; Range = 1–5) for each item in both groups (group 1; group 2). No significant differences were registered between groups testing the SG at different implementation levels as detected by the Mann-Whitney nonparametric test. Then, although the complexity of the SG increased from baseline to the prototype version, participants always found the SG usable. Data of items 1–10 are reported in Table 3.

**Table 3:** Summary statistics for each item of the *ad-hoc* questionnaire on SG functionalities per group.

**Item**	**Group 1**			**Group 2**		
	***N***	**Median**	**25–75 P**	***N***	**Median**	**25–75 P**
**1**	10	5.00	4.00–5.00	28	4.00	4.00–4.50
**2**	10	5.00	4.00–5.00	28	4.00	4.00–5.00
**3**	10	5.00	4.00–5.00	28	4.00	4.00–5.00
**4**	10	3.50	3.00–4.00	28	3.50	2.00–4.00
**5**	10	4.00	4.00–5.00	28	4.00	3.50–5.00
**6**	10	3.50	2.00–4.00	28	4.00	3.00–4.00
**7**	10	4.00	4.00–5.00	28	4.00	4.00–4.50
**8**	10	4.00	4.00–5.00	28	4.00	4.00–4.00
**9**	10	4.00	4.00–5.00	28	4.00	4.00–4.00
**10**	10	4.00	4.00–4.00	28	4.00	3.00–4.00

**Fig. 1:**
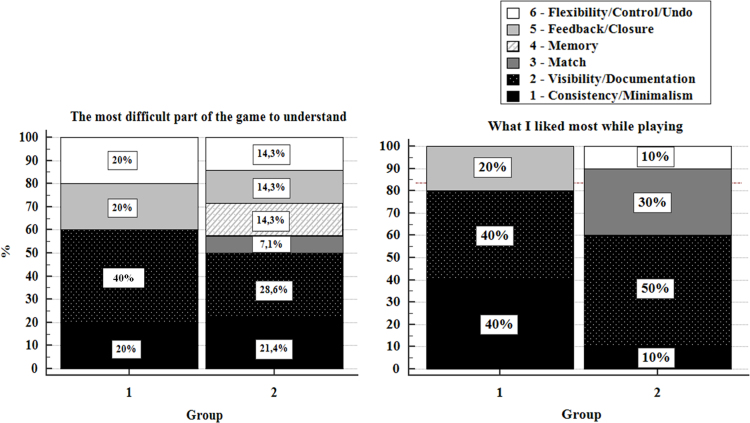
Percentage of answers to the *ad hoc* questionnaire on SG functionalities per usability principle per group (group 1; group 2). On the left, answers to the question “What was the most difficult part to understand?” (item 11); on the right, answers to the question “What did you like the most while playing the game?” (item 12).

Percentages of responses for each of the two open-ended questions of the questionnaire on functionalities are reported in Fig. 1. Considering the most difficult part of the SG to understand while playing (item 11), the majority of participants of group 1 identified as a problem the visibility of information to interact with the SG (Visibility/Documentation). At the same time, the consistency of features throughout the game (Consistency/Minimalism), the possibility to have a feedback (Feedback/Closure) and a smooth and flow interaction with the game (Flexibility/Control/Undo) were considered as improvable. Functionalities at this level of implementation of the SG were very low and these elements were still to be strengthened. Participants of group 2 found an improvement in the visibility and informativeness of the SG (Visibility/Documentation), and also in the feedback about users’ actions and performance (Feedback/Closure), and the flexibility of the SG interaction (Flexibility/Control/Undo).

When the information on clinical scales was added to the SG to improve learning challenge (second release, group 2), the need for users to memorize information (Memory) was perceived as a difficulty. In addition, in the second release when the environment changed from a standard gym to a rehabilitation gym, the match between the SG and real world was considered a difficult part of the SG by a small part of participants (Match). Nevertheless, the 30% of the participant found the match between the SG rehabilitation gym and real world as the most likable part of the game. The consistency of SG features throughout the game (Consistency/Minimalism) obtained a high percentage of preferences in the first release. This data decreased in the second release in favor of different aspects of the game (Visibility/Documentation; Match; Flexibility/Control/Undo) showing a higher differentiation of its functionalities.

### 2.5 Game modifications

In Table 4, we reported the characteristics of the SG at baseline and in the next two releases of the game per each usability principle defined above (Table 3). These data are used for the validation of the Serious Game presented in Savazzi et al. [1].

**Table 4:** Characteristics of the SG along implementation releases.

**Usability principles**^a^	**SG first release**	**SG second release**	**SG prototype**
***Consistency/Minimalism***	The layout of the game was basic.	The layout of the game changed homologating colors and fonts.	Colors and fonts were homologated throughout the game.
	A few information on the SG was provided.	A single page was presented with all the instructions and information on the SG.	Successive pages were presented with specific information for each step of the game.
	Limited number of ways of interaction with the environment:	Increased number of ways of interaction with the environment:	Increased number of ways of interaction with the environment:
	• Rotating, • Selecting, • Moving	• Rotating, • Selecting, • Moving, • Setting rehabilitative activities, • Reviewing clinical scales indications and cut-off.	• Rotating, • Selecting, • Moving, • Setting rehabilitative activities, • Reviewing clinical scales indications and cut-offs, • Check and modify parameters of rehabilitative activities (such as speed of treadmill and duration of treatment).
***Visibility/Documentation***	A text message with game instruction appeared.	A text message with game instruction was included in each step of the SG.	A text message with game instruction appeared in each step of the SG.
			The size and color of the font favored good visibility of the text.
		The size of the font was adjusted for a better visibility.	
		The colors of the text were modified for a better figure-ground contrast.	
***Match***	The avatar was a young female.	The avatar became an elderly man patient.	Three different patient׳s avatars were included, their aspect was representative of three different pathologies.
	The environment was a gym.	The environment was a rehabilitation gym with a limited number of items.	The environment was a rehabilitation gym with an increased number of tools for rehabilitation activities.
			When the user chose a rehabilitation procedure for the patient, the avatar started to perform the clinical exercise prescribed.
***Memory***	Clinical information of the patient was included in the clinical chart.	Clinical information of the patient was included in the clinical chart.	The number of clinical case passed from 1 to 3.
			Clinical information of patient is included in the clinical charts.
		Information on clinical scales was added to support clinical chart interpretation.	
			Information on clinical scales were added to support clinical chart interpretation.
***Feedback/Closure***	No feedback was included.	The total score of the game appeared at the end of the game.	The total score of the game appeared at the end of the game.
			Immediate feedback message appeared at the end of the game: “Very Good” or “Continue improving by playing”
***Flexibility/Control/Undo***	Very low flexibility.	The possibility to stop the game was added.	The possibility to stop the game was included.
		More options of patient׳s rehabilitation activities were made available.	Many options of patient׳s rehabilitation activities were available.
***Error/Message***	–	–	–
***Language***	The text included in the game was in English language.	The text included in the game was in Italian language and was reviewed in order to be clear.	The text included in the game was in clear Italian language.

a Based on Nielsen–Shneiderman usability heuristics adapted from Zhang and Walji [2].

## References

[1] Savazzi, Isernia S., Jonsdottir J., Di Tella S., Pazzi S., Baglio F. (2018). Engaged in learning neurorehabilitation: development and validation of a serious game with user-centered design. Comput. Educ..

[2] Zhang J., Walji M.F. (2011). TURF Toward a unified framework of EHR usability. J. Biomed. Inform..

[3] Nielsen Jakob, Mack R.L. (1994). Usability Inspection Methods.

[4] Shneiderman Ben (1998). Designing the User Interface, Strategies for Effective Human Computer Interaction.

