# Supplementary material for: Design and implementation of a Serious Game on neurorehabilitation: Data on modifications of functionalities along implementation releases
Source: Data Brief. 2018 Aug 30;20:864–9. doi: 10.1016/j.dib.2018.08.100 (PMC6134168; doi:10.1016/j.dib.2018.08.100)
Supplement: Supplementary file 1 — Supplementary material [file mmc1.pdf]

Conflicts of Interest Statement

Manuscript title: DESIGN AND IMPLEMENTATION OF A SERIOUS GAME ON NEUROREHABILITATION: DATA ON MODIFICATIONS OF FUNCTIONLITIES ALONG IMPLEMENTATION RELEASES

The authors whose names are listed immediately below certify that they have NO affiliations with or involvement in any organization or entity with any financial interest (such as honoraria; educational grants; participation in speakers' bureaus; membership, employment, consultancies, stock ownership, or other equity interest; and expert testimony or patent-licensing arrangements), or non-financial interest (such as personal or professional relationships, affiliations, knowledge or beliefs) in the subject matter or materials discussed in this manuscript.

Author names:

FEDERICA SAVAZZI

SARA ISERNIA

JOHANNA JONSDOTTIR

SONIA DI TELLA

STEFANIA PAZZI

FRANCESCA BAGLIO

The authors whose names are listed immediately below report the following details of affiliation or involvement in an organization or entity with a financial or non-financial interest in the subject matter or materials discussed in this manuscript. Please specify the nature of the conflict on a separate sheet of paper if the space below is inadequate.

Author names:

This statement is signed by all the authors to indicate agreement that the above information is true and correct (*a photocopy of this form may be used if there are more than 10 authors*):

Author's name (typed)	Author's signature	Date
FEDERICA SAVAZZI<br>FEDERICA SAVAZZI	[illegible]	06/06/2018
SARA ISERNIA<br>SARA ISERNIA	[illegible]	06/06/2018
JOHANNA JONSDOTTIR<br>JOHANNA JONSDOTTIR	Johanna Jonsdott	06/06/2018
SONIA DI TELLA<br>SONIA DI TELLA	Sonia Di Tella	06/06/2018
STEFANIA PAZZI<br>STEFANIA PAZZI	[illegible]	07/06/2018
FRANCESCA BAGLIO<br>FRANCESCA BAGLIO	Francesca Baglio	06/06/2018
